# The 1-monolaurin inhibit growth and eradicate the biofilm formed by clinical isolates of *Staphylococcus epidermidis*

**DOI:** 10.1186/s12919-019-0174-9

**Published:** 2019-12-16

**Authors:** Andre Krislee, Chaerul Fadly, Dwi Aris Agung Nugrahaningsih, Titik Nuryastuti, Febri Odel Nitbani, Jumina, Eti Nurwening Sholikhah

**Affiliations:** 1 Faculty of Medicine, Public Health, and Nursing, Universitas Gadjah Mada, Yogyakarta, Indonesia; 2 Departement of Pharmacology and Therapy Faculty of Medicine, Public Health, and Nursing, Universitas Gadjah Mada, Yogyakarta, Indonesia; 3 Departement Microbiology, Faculty of Medicine, Public Health, and Nursing, Universitas Gadjah Mada, Yogyakarta, Indonesia; 4 Departement of Chemistry, Faculty of Science and Engineering, Universitas Nusa Cendana, Kupang, Indonesia; 5 Departement of Chemistry, Faculty of Mathematics and Natural Sciences, Universitas Gadjah Mada, Yogyakarta, Indonesia

**Keywords:** Biofilm, *Staphylococcus epidermidis*, 1-monolaurin, MIC, MBC, BIC, BEC

## Abstract

**Background:**

Biofilm is one of the causes of antibiotic resistance. One of the biofilm-producing bacteria is *Staphylococcus epidermidis* which has been proven to infect long-term users of urinary catheters and implant devices. The 1-monolaurin compound has been known to have an antimicrobial effect. However, its effect on clinical isolates of *S. epidermidis* in producing biofilm has not been established. This study was conducted to investigate the effect of 1-monolaurin towards biofilm forming clinical isolates of *S. epidermidis*.

**Methods:**

The experiment used micro broth dilution technique which consists of test group (1-monolaurin), positive control group (rifampicin), solvent group, negative control group (clinical isolate of *S. epidermidis*), and media group (TSB media). The Minimal Inhibition Concentration (MIC) was determined by incubating bacteria added with 1-monolaurin (1000–1953 μg/mL) or rifampicin (250–0,488 μg/mL) for 24 h. The MIC was determined visually. After that, the incubated bacteria was cultured in TSA media to determine Minimal Bactericidal Concentration (MBC). The assessment of Biofilm inhibitory Concentration (BIC) and Biofilm Eradication Concentration (BEC) was conducted with the same way, the difference was BIC intervened directly with compound meanwhile BEC was incubated for 24 h in 37 °C before the intervention. Then, the specimen was reincubated to grow biofilm at the microplate, washed with PBS and stained with 1% of crystal violet. The optical density (OD) was measured at a wavelength of 595 nm. The percentage of BIC and BEC then were calculated, continued to probit analysis regression to determine the BIC50, BIC80, BEC50, and BEC80.

**Results:**

The MIC dan MBC of 1-monolaurin and rifampicin were > 1000 μg/mL, > 1000 μg/mL, ≤0.488 μg/mL, and 1.953 μg/mL respectively. BIC50 and BIC80 of 1-monolaurin and rifampicin were 26.669 μg/mL, 168.688 μg/mL, 0.079 μg/mL, and 0.974 μg/mL respectively. The BEC50 and BEC80 of 1-monolaurin and rifampicin were 322.504 μg/mL, 1338.681 μg/mL, 5.547 μg/mL, dan 17.910 μg/mL respectively.

**Conclusion:**

The 1-monolaurin can inhibit growth and eradicate the biofilm formed by clinical isolates of *S. epidermidis*, however, it has neither inhibit nor kill planktonic cells of *S. epidermidis.*

## Background

Naturally, microorganisms attach to and grow in living and inanimate surface, such as enamel, cardiac valve, lung, middle ear as well as medical devices. The appearance of microorganisms growth that often occurs is biofilm formation. Microorganism produces Extracellular Polymeric Substance (EPS) that facilitate attachment and biofilm formation as a result change host phenotype. Biofilm has become a serious health problem because of the increased resistance to antibacterial and its potential to cause infection in patients using medical equipment. There are at least three reasons why biofilms can cause antibiotic resistance: (1) antibiotic agents diffuse into EPS matrix and become inactive, (2) biofilms reduce microorganism growth rates that affect antibiotic inactivation, and (3) the environment around cells protects the organism, such as decrease antibiotic uptake into cells [1].

Biofilm formation process through five stages. The first stage includes an initial attachment that can occur actively or passively. This process depends on the physicochemical components of bacteria and their surface components. At this stage, the bacteria still inherently reversible. Furthermore, the bacteria will attach irreversibly. In this second stage, the release of biofilms attachment requires strong strengths such as detergent, surfactant, sanitizer and/or heating. The third stage has entered the initial process of establishing an architecture of biofilm (microcolony formation). Microcolony formation resulted from the accumulation and growth of microorganisms and the production of EPS. This strengthens the bacterial bond with the host. Then, it will enter the biofilm maturation stage, the fourth stage, which develope at least 10 days or more. The last stage is the dispersion stage. At this stage, bacterial cells will return to their planktonic cells and come out of the biofilm to form new colonies [2].

Some microorganisms that can form biofilms are gram-positive bacteria, such as *Staphylococcus aureus* and *Staphylococcus epidermidis*, gram-negative bacteria including *Pseudomonas aeruginosa*, *Escherichia coli* and several genus Candida especially *Candida albicans* and *Candida tropicalis* [3]. One of the species will be discussed here is *S. epidermidis* which is a gram-positive bacteria coagulase-negative staphylococci group [4].

*Staphylococcus epidermidis* is a commensal bacteria that colonize in the skin and mucous membranes of humans and other mammals. The colony of *S. epidermidis* predominantly in axillae, head, and nares. As science develops, *S. epidermidis* has been proven to often contaminate medical devices, especially in peripheral and central catheter placement. Besides, these bacteria play a role in infection of prosthetic joints, vascular grafting, surgery, cranial nerve system shunts, and cardiac devices [5].

The mechanism of *S. epidermidis* in forming biofilms is through the biochemical and molecular process. Polysaccharides adhesin have an important role in this biochemical process. The two main polysaccharides produced by *S. epidermidis* are capsular polysaccharide adhesin (PSA) and polysaccharide intercellular adhesin (PIA). The PSA plays a role in initiation attachment and PIA play role in cell accumulation. The PIA itself is coded by the intercellular adhesin (ica) gene [6]. More than 85% of *S. epidermidis* isolated bacteria from blood cultures of the patient in hospitals have the ica gene [7]. In Addition, the *S. epidermidis* was the third main bacteria at Fatmawati Hospital that often obtained from the culture of patients entering the Intensive Care Unit (ICU) after *P. aeruginosa* and *K. Pneumonia* [8]. Therefore antibiotics against *S. epidermidis* especially the clinical isolate is needed.

Natural compounds are known to be potential for new antibiotic [9]. One of the natural compounds that have been shown have an antibacterial activity is 1-monolaurin. The 1-monolaurin is a compound derived from coconut oil. Some bacteria that have been proven to be inactivated by monolaurin are *Liseteria monocytogenes*, *Helicobacter pylori*, *Hemophilus influenza*, *Staphylococcus aureus*, *Streptococcus* groups A, B, F, and G [10]. However, the antibacterial and antibiofilm activity, especially inhibition and bactericidal of planktonic cells and inhibition and eradication of biofilms from 1-monolaurin against clinical isolates of *S. epidermidis* is unknown.

## Materials and methods

### Materials

The 1-monolaurin was obtained from Nitbani [11]. The isolates of *S. epidermidis* obtained from the collection of Microbiology Laboratory Faculty of Medicine, Public Health, and Nursing UGM. The Dimethyl Sulfoxide (DMSO), NaCl, violet crystal, 96% of ethanol, Phosphate Buffered Saline (PBS) with pH of 7.4, TSB media, and TSA media were obtained from Microbiology Laboratory inventory, Faculty of Medicine, Public Health, and Nursing UGM. The 96-well microplate with a flat-shaped base from Biosigma, Italian and U-shaped base from Iwaki, Japan.

## Methods

### Preparing 1-monolaurin

The 1-monolaurin was prepared by mixing 2 mg with 50 μg/mL of pure DMSO and 950 μg/mL TSB media then being vortex to produce 1-monolaurin dissolved in 5% of DMSO as stock solution. The various concentration of 1-monolaurin was made from this stock solution.

### Preparing clinical isolate of *Staphylococcus epidermidis*

The clinical isolate of bacteria producing biofilm *S. epidermidis* was prepared in suspension by mixing the pellets of *S. epidermidis* clinical isolates with 0.9% sterile NaCl. The clarity of the mixture was compared with McFarland 0.5. Furthermore, the suspension was diluted with TSB media with a ratio of 1: 100. The bacterial suspension was prepared in a concentration of 1 × 10^6^ CFU/mL.

### Minimum inhibitory concentration and minimum bactericidal concentration assay

The Minimum inhibitory concentration (MIC) and Minimum Bactericidal Concentration (MBC) assay were conducted using micro broth dilution assay [12]. The MIC is the lowest level of the compound which can inhibit the growth of bacterial planktonic cells, while MBC is the lowest level of a compound that can kill 99.9% of bacterial planktonic cells. The 1-monolaurin or rifampicin at various concentration were filled triplicate to each well of a flat-shaped microplate, and the same volume of suspension of *S. epidermidis* clinical isolates were added. The final concentration of 1-monolaurin in the plate was 1000–1.953 μg/mL and 250–0.488 μg/mL for rifampicin. After 24 h incubation at 37 °C, MIC was determined visually by observing the presence or absence of planktonic cell growth. The final MIC value is the mode value of the MIC in each well. The MBC was determined by adding 10 μL of liquid from a clear well to the TSA media, after 24 h incubation at 37 °C by observing whether there was bacterial growth in TSA media.

### Biofilm inhibitory concentration assay

Biofilm inhibitory Concentration (BIC) assay was conducted by microtiter plate assay [13]. Biofilm inhibitory testing used a microplate with a U-shaped base with the volume in each well was 100 μL. Biofilm testing procedures have the same procedures with planktonic cell testing, the difference was after microplates were incubated, microplates were washed with PBS to separate the formed biofilm, and was given 1% of crystal violet, then washed again with PBS and finally was given 96% of alcohol and left for 15 min. All experiments were carried out in triplicate. The Optical Density (OD) was measured at a wavelength of 595 nm. The percentage of biofilm inhibitory was calculated using the following formula: [(OD growth control – OD sample) / OD growth control] × 100 [14]. Then, the biofilm formation inhibition such as BIC50 and BIC80 were determined by probit analysis regression [15].

### Biofilm eradication concentration assay

Biofilm Eradication Concentration (BEC) Assay was conducted by the same procedures as the BIC assay. The biofilm eradication testing was started by growing the biofilm first by incubating the suspension of *S. epidermidis* clinical isolates for 24 h at 37 °C. Then, each well of microplate was washed with PBS with pH of 7.4 so that it leaves only the biofilm and the 1-monolaurin or rifampicin with various concentration was added. After that, the microplate was incubated for 24 h at 37 °C and was washed with PBS, add 1% of crystal violet and was washed again with PBS and finally 96% alcohol was given and left for 15 min. All experiments were conducted in triplicate and three data were generated in each experiment. The Optical Density (OD) was measured at a wavelength of 595 nm. The percentage of biofilm eradication was calculated using the following formula: [(OD growth control – OD sample) / OD growth control] × 100 [14]. Then, the biofilm formation eradication such as BEC50 dan BEC80 were determined by probit analysis regression [15].

## Result

### The minimum inhibitory concentration (MIC) and minimum bactericidal concentration (MBC)

Table 1 showed the MIC and MBC of 1-monolaurin or rifampicin on planktonic cell of *S. epidermidis* clinical isolate.

**Table 1:** The MIC and MBC of 1-monolaurin or rifampicin on planktonic cell of *S. epidermidis* clinical isolate

Compounds	MIC (μg/mL)^a^	MBC (μg/mL)
1-monolaurin	> 1000	> 1000
Rifampicin	≤0.488	1.953

^a^mode concentration of testing compound in triplicate test

### The biofilm inhibitory concentration (BIC)

Biofilm growth inhibition testing of *S. epidermidis* clinical isolates described in BIC50 and BIC80 that was obtained from probit regression analysis shown in Table 2.

**Table 2:** The inhibition biofilm formation of 1-monolaurin or rifampicin on *S. epidermidis* clinical isolates

*S. epidermidis* clinical isolate	TSB Media	Compound	Concentration (μg/mL)	OD ± SD			BIC (%)	BIC50 (μg/mL)	BIC80 (μg/mL)
+	+	O	–	0.454	±	0.040	0	–	–
O	+	O	–	0.081	±	0.003	100	–	–
+	+	5% of DMSO	–	0.456	±	0.020	−0.537	–	–
+	+	1-monolaurin	1000	0.071	±	0.032	102.773	26.669	166.688
			500	0.093	±	0.040	96.780		
			250	0.163	±	0.013	77.996		
			125	0.240	±	0.200	57.424		
			62.5	0.226	±	0.194	61.181		
			31.25	0.248	±	0.025	55.277		
			15.625	0.288	±	0.024	44.365		
			7.813	0.362	±	0.036	24.597		
			3.906	0.375	±	0.033	21.109		
			1.953	0.375	±	0.013	21.119		
+	+	Rifampicin	250	0.075	±	0.003	101.699	0.079	0.974
			125	0.090	±	0.009	97.764		
			62.5	0.098	±	0.008	95.349		
			31.25	0.094	±	0.001	96.601		
			15.625	0.105	±	0.007	93.560		
			7.813	0.094	±	0.001	96.422		
			3.906	0.099	±	0.009	95.170		
			1.953	0.116	±	0.008	90.698		
			0.977	0.191	±	0.050	70.483		
			0.488	0.188	±	0.011	71.288		

### The biofilm eradication concentration (BEC)

The eradication biofilm formation activity of 1-monolaurin or rifampicin on *S. epidermidis* clinical isolates was presented in Table 3. The BEC50 and BEC80 were obtained by probit regression analysis.

**Table 3:** The eradication biofilm formation of 1-monolaurin or rifampicin on *S. epidermidis* clinical isolates

*S. epidermidis* clinical isolate	TSB Media	Compound	Concentration (μg/mL)	OD ± SD		BEC (%)		BEC50 (μg/mL)	BEC80 (μg/mL)
+	+	O	–	0.463	±	0.041	0	–	–
O	+	O	–	0.106	±	0.006	100	–	–
+	+	DMSO 5%	–	0.476	±	0.090	−3.641	–	–
+	+	1-monolaurin	1000	0.165	**±**	0.018	83.380	322.504	1338.681
			500	0.219	**±**	0.039	68.161		
			250	0.363	**±**	0.064	27.918		
			125	0.398	**±**	0.033	18.114		
			62.5	0.372	**±**	0.070	25.397		
			31.25	0.423	**±**	0.082	11.111		
			15.625	0.433	**±**	0.064	8.403		
			7.813	0.465	**±**	0.077	−0.654		
			3.906	0.465	**±**	0.038	−0.654		
			1.953	0.392	**±**	0.082	19.701		
+	+	Rifampicin	250	0.118	±	0.010	96.452	5.547	17.910
			125	0.114	±	0.011	97.666		
			62.5	0.107	±	0.002	99.720		
			31.25	0.123	±	0.011	95.238		
			15.625	0.204	±	0.016	77.362		
			7.813	0.221	±	0.052	67.787		
			3.906	0.278	±	0.060	51.727		
			1.953	0.394	±	0.100	19.328		
			0.977	0.450	±	0.105	3.548		
			0.488	0.466	±	0.121	2.708		

## Discussion

The 1-monolaurin cannot inhibit growth and kill planktonic cells of *S. epidermidis* clinical isolates at the highest concentrations tested. The rifampicin as a positive control, has been shown to have the effect of inhibiting growth and killing bacterial planktonic cells. This results in accordance with the reference of Clinical & Laboratory Standards Institute [16]. In this this study, the MIC and MBC of rifampicin ​​for planktonic cells of *S. epidermidis* clinical isolates were ≤ 0.488 μg/mL and 1.953 μg/mL. According to the CLSI [12], the *S. epidermidis* clinical isolate which used in this study showed its sensitivity to antibiotics. The solvent of 1-monolaurin compound used, 5% of dimethylsulphoxide, did not show any effect on planktonic or biofilm cells. Other study using 10% of dimethylsulphoxide also showed that the 10% of dimethylsulphoxide did not show any effect on bacterial growth [14].

In contrast with testing on planktonic cell, both the 1-monolaurin and rifampicin have activity in inhibiting growth and eradicate the biofilm formation of *S. epidermidis* clinical isolate. As positive control in this study, the BIC50 and BIC80 of rifampicin were 0.079 μg/mL and 0.974 μg/mL. These results were not different from previous studies that showed rifampicin had the effect of inhibiting biofilm formation at concentrations < 0.0625 μg/mL [17]. The BEC50 and BEC80 rifampicin were 5.547 μg/mL and 17.910 μg/mL. These results were not different from the study conducted by Laverty [18] which showed that rifampicin could eradicate biofilms at concentrations of 62.5 μg/mL. This supports the Marquez [19] study which shows that rifampicin has a higher sensitivity to *S. epidermidis* compared to some antibiotics such as vancomycin, ceftaroline, erythromycin, fusidic acid, gentamicin, linezolid, and pristinamisin.

The minimum inhibitory concentration (MIC) and minimum bactericidal concentration (MBC) of 1-monolaurin on *S. epidermidis* clinical isoloate was > 1000 μg/mL. From the results of the previous studies [11] showed that 1-monolaurin can inhibit the formation of planktonic cells from other Staphylococcus groups, *S. aureus* at a concentration of 500 μg/ mL. Besides, a study conducted by Tangwathcharin [20] showed that compound 1-monolaurin required a concentration of 100 μg/mL to kill *S. aureus* planktonic cells and their clinical isolates. *Staphylococcus epidermidis* has been shown to have a higher resistance to antibiotics than *S. aureus* [21].

Referring to Holetz [22] study, the compound with concentrations more than 1000 μg / mL did not have antimicrobial effects. This shows that 1-monolaurin does not have the effect of inhibiting or killing planktonic cells of *S. epidermidis* clinical isolates.

The results showed that 1-monolaurin can inhibit biofilm formation of *S. epidermidis* clinical isolates. The BIC50 and BIC80 1-monolaurin values ​​were 26.669 μg/mL and 168.688 μg/mL. The 1-monolaurin can inhibit the formation of biofilms by reducing the hydrophobicity of bacterial cells and preventing attachment of bacterial cells [23]. If the bacteria is too hydrophobic or hydrophilic it can cause damage to the biofilm structure [24]. The inhibitory effect was similar to the Schlievert [25] study which showed monolaurin had a 66% inhibitory effect on *S. aureus* biofilm at a concentration of 48 μg/mL monolaurin. Besides, monolaurin can inhibit biofilm formation in other bacteria such as *S. mutans* which is the main bacterium on human dental plaques at a concentration of 95 μg/mL [23].

The results showed that 1-monolaurin can eradicate the formation of biofilm *S. epidermidis* clinical isolate. The BEC50 and BEC80 1-monolaurin values ​​were 322.504 μg/mL and 1338.681 μg/mL. Its seems like the Goc [26] study which showed that monolaurin can eradicate 50% of biofilm formation from *Borrelia sp.* at a concentration of 375 μg/mL. From previous studies, it was found that monolaurin can change the morphological structure of biofilms which are similar to proteolytic enzyme activity (proteases and phospholipases) [27].

The interesting thing to discuss is that 1-monolaurin requires a higher concentration for inhibit and kill the planktonic cells of *S. epidermidis* clinical isolates than inhibit and eradicate *S. epidermidis* biofilm isolates. This is different from the Donlan^1^ study which showed that biofilms increased antimicrobial resistance. Compared to monolaurin studies on *Borellia sp.*, it also shows the MIC and MBC values ​​which are lower than the BEC50 value [25]. The explanation of fact in our research is 1-monolaurin compound needs higher effort or higher concentration when it interacts with cell wall of the planktonic cells of *S. epidermidis* clinical isolates. *S. epidermidis* is Gram positive bacteria that have several layers of peptidoglycan in its cell wall. Therefore, 1-monolaurin with higher concentration is needed to destroy the cell wall of *S. epidermidis*. In contrast, a biofilm isolates of *S. epidermidis* is a substance (single substance) produced by this bacteria to protect its self or its coloni. So, 1-monolaurin as an antibiofilm agent is needed in a slower concentration to interact with biofilm *S. epidermidis.* 1-Monolaurin compound shows its high ability as antibiofim to inhibit and kill *S. epidermidis* biofilm isolates because its have a lauril group (lipophilic side) and 2 hydroxyl group (hydrophilic side). The two different groups in monolaurin structure can interact with the lipophilic and the hydrophobic substance in *S. epidermidis* biofilm isolates through Hydrogen and Van de Waals interaction.

## Conclusions

The 1-monolaurin can inhibit growth and eradicate the biofilm formed by clinical isolates of *S. epidermidis*, however it has neither inhibit nor kill planktonic cells of *S. epidermidis.* These findings showed that the 1-monolaurin potential as antibiotics against clinical isolates of *S. epidermidis.*

## Data Availability

The data used to support the findings of this study are available from the corresponding author upon request.

## Abbreviations

BEC: Biofilm eradication concentration
BIC: Biofilm inhibitory concentration
CFU/mL: Colony-forming units per mililiter
CLSI: Clinical & laboratory standards institute
DMSO: Dimethyl sulfoxide
EPS: Extracellular polymeric substance
ICU: Intensive care unit
MBC: Minimal bactericidal concentration
MIC: Minimal inhibition concentration
OD: Optical density
PBS: Phosphate buffered saline
PIA: Polysaccharide intercellular adhesin
PSA: Polysaccharide adhesin
TSA: Tryptic soy agar
TSB: Tryptic soy broth
